# Collaborative optimization model and algorithm for airport capacity and traffic flow allocation

**DOI:** 10.1371/journal.pone.0298540

**Published:** 2024-03-22

**Authors:** Peinan He, Weijun Pan

**Affiliations:** College of Air Traffic Management, Civil Aviation Flight University of China, Guanghan, Sichuan, China; Southwest Jiaotong University, CHINA

## Abstract

How to efficiently utilize the existing airport capacity without physical expansion and considerable economic inputs to meet air traffic needs is one of the important tasks of air traffic management. To improve the efficiency of capacity utilization, it is necessary to find the actual airport capacity properly. In this work, taking Shuangliu International Airport as an example, a methodology for capacity estimation is proposed that combines the empirical method with an analytical approach that uses historical performance data from the airport to construct a capacity envelope to approximate the airport’s actual capacity to the greatest extent, establishes a collaborative optimization model that reflects the inherent relations between airport capacity and arrival and departure traffic demand, adopts an improved optimization algorithm to solve the model, and generates an optimal flight allocation scheme. Priority ratio is introduced to dynamically adjust management preferences for arrival and departure traffic demand to further reveal the synergy mechanism between departure and arrival traffic flow demand and the airport capacity. The result shows that the Flight On-time Performance rate is lifted by 6% in the case study which proves the feasibility of the proposed method, demonstrating its value for maximizing airport capacity and traffic flow demand without requiring expansions on airport scales.

## 1. Introduction

Air traffic congestion and delays caused by insufficient airport capacity and increased traffic demand have become a focus of attention in the global civil aviation industry. The growing demand for air travel, combined with the rapid development of the logistics industry, has resulted in a significant increase in air traffic flow. However, flight delays and air traffic congestion have increased in frequency. In the long run, as it makes airport and air traffic management more challenging, lowers customer satisfaction with travel experiences, and brings economic losses to airlines accordingly, it will undoubtedly have an impact on the sustainable growth of the aviation industry. The situation exists not only in China, but also in other countries and regions with developed air transportation industries around the world. Many researchers and research institutions [1–5] have begun to investigate ways to increase airport capacity to meet traffic demand, as well as solutions to distribute airport capacity and traffic demand, with the goal of exploring effective methods to reduce traffic congestion and delays.

In this paper, aiming to effectively utilize existing capacity resources to meet traffic demand without expanding the airport’s existing scale, the authors proposed an optimized analytical approach combined with an empirical method to estimate an airport’s practical capacity and establish a model that reflects the collaborative response between airport capacity and arrivals and departure demand (in Section 3). And a developed optimization algorithm presented (in Section 4) to solve the problem, using historical operational data from Chengdu Shuangliu International Airport as sample sets to validate the method.

## 2. Background

Problems with airport capacity and flight capacity demand, air traffic congestion, and flight delays have remained highly concerning over the last few decades. In the early 60s, the Federal Aviation Administration (FAA) and Airborne Instrumentation Laboratory (AIL) [5, 6] described a method to estimate the runway capacity under various runway configuration, and the corresponding delays at different demand levels. A review by G. F. Newell [7] provided a principle of runway design considering the effects of runway geometry, flying regulations, and aircraft sequencing on runway capacity. A few prior studies estimated the mean inter-operation times by accounting for several factors, including randomness in speed, variations in runway occupancy times, uncertainty in the aircraft fleet combination, and uncertainty in the time of aircraft appearance at specific points at different phases of arrival and departure. Eugene P. Gilbo [4–8] proposed a ground capacity prediction model for optimizing airport capacity utilization, which laid the foundation for collaboratively scheduling arrivals and departures. In recent years, Alain Urbeltz Isla and Hildoberto Augusto de Oliveira [9] believed that one of the main processes to ensure airport operational efficiency must count on flight schedule management and coordination. Joeri Aulman [10] revealed that the use of real-time operational data and predictive planning can help optimize airport capacity. Mayara Condé Rocha Murça and John Hansman [11] developed an optimization model for capacity allocation that was considered to be superior to many methods for predicting the throughput performance of New York Airport. Katsigiannis Fotios A. and Zografos Konstantinos G. [12] suggested a dynamic slot allocation model for airport resources management and a solution framework for improving airport slot schedules and the utilization of airport capacity. Based on these aforementioned contributing works and research, it is evident that for capacity estimation, using empirical method along with an analytical approach with real-time practical data for further correction is expected to produce a more trustworthy estimation.

Chengdu Shuangliu International Airport is one of the busiest airports in the world, rated 4F, with two parallel runways for civil transport use. It is also ranked as one of the largest hub airports in China, serving more than 300 domestic and international routes. The total number of inbound and outbound flights at Chengdu Shuangliu International Airport in 2019 was 366887, with an average of 1005 flights per day, according to the 2019 Civil Aviation Airport Production Statistics Bulletin [13]. It had 1007 flights per day, as reported in the Civil Aviation Air Traffic Control Operation Statistics Report for the First Half of 2020 [14]. However, traffic demand is increasing every year, while the risk of traffic congestion and flight delays significantly increases. In recent years, the demand for traffic has been continuously rising, and the imbalance between supply and demand has become more prominent. Therefore, using this airport as an example for capacity estimation research is an attempt to help the airport with capacity resource utilization by presenting the suggested method in this work without considering spending fortunes on expansions. The results will serve as a reference for the airport and air traffic flow management parties of other busy airports that are similar to it.

## 3. Methods

### 3.1 Capacity estimation

The empirical process of building a capacity envelope is predicated on the idea that, for a given time period, the observed peak arrival and departure counts represent both the airport’s maximum operating capacity and its performance at or near capacity. It is reasonable to assume that the historical peak data for those airports represents their maximum operational capabilities and can be useful in estimating capacity. In addition, when there is more demand for flights than there is capacity, congestion frequently results. The purpose of choosing peak days for research is to find out if the suggested approach can be used to get rid of or lessen spillover flights that occur when demand exceeds the capacity limit during periods of extreme traffic flow. If the proposed method counts, the airport’s actual capacity can be increased even further, and delays can theoretically be prevented during any other time when there is little demand for flights.

The peak transportation season at the airport is in the summer and autumn. Thus, after a long period of observation, this study collected practical operation data from Chengdu Shuangliu International Airport in the summer and autumn, and created a research sample dataset. As an example, for demonstration in the following sections and parts, this work particularly presents historical airport performance data for August 19 2020, which was the day with the most flights taking off and landing this season. According to the observation, it was the busiest day at the airport, with the longest duration, lasting 17 hours (from 07:00 to 24:00). It is confirmed that the data covers a very wide range, with values encompassing all other peak-day data. Therefore, this data listed in Table 1 used in this research is representative and reliable.

**Table 1:** Historical airport performance data.

Slot /15min		Initial traffic demand and delay				Total
No.	Start End	Departure	Cancellation	Arrival	Cancellation	/hour
1	0:00–0:15	3		12		48
2	0:15–0:30	0		11		
3	0:30–0:45	1		11		
4	0:45–1:00	1		9		
5	1:00–1:15	0		7	1	40
6	1:15–1:30	0		11		
7	1:30–1:45	1		8		
8	1:45–2:00	3		10		
9	2:00–2:15	0		8		15
10	2:15–2:30	1	1	2		
11	2:30–2:45	1		0		
12	2:45–3:00	3		0		
13	3:00–3:15	2		0		4
14	3:15–3:30	2		0		
15	3:30–3:45	0		0		
16	3:45–4:00	0		0		
17	4:00–4:15	0		1		3
18	4:15–4:30	0		2		
19	4:30–4:45	0		0		
20	4:45–5:00	0		0		
21	5:00–5:15	0		2		18
22	5:15–5:30	2		1		
23	5:30–5:45	2		1		
24	5:45–6:00	8		2		
25	6:00–6:15	7		1		43
26	6:15–6:30	13		0		
27	6:30–6:45	10		1		
28	6:45–7:00	11		0		
29	7:00–7:15	11	2	1		48
30	7:15–7:30	11	2	1		
31	7:30–7:45	12		1		
32	7:45–8:00	9		2		
33	8:00–8:15	11		1		50
34	8:15–8:30	14	1	0		
35	8:30–8:45	8		4		
36	8:45–9:00	7		5		
37	9:00–9:15	9	1	3		50
38	9:15–9:30	10	1	3		
39	9:30–9:45	7		5	1	
40	9:45–10:00	8		5	2	
41	10:00–10:15	6		5		53
42	10:15–10:30	4		10		
43	10:30–10:45	4	2	7		
44	10:45–11:00	8		9		
45	11:00–11:15	3		7		52
46	11:15–11:30	8		6		
47	11:30–11:45	6		6		
48	11:45–12:00	14		2		
49	12:00–12:15	5		4		54
50	12:15–12:30	7		6		
51	12:30–12:45	5		9		
52	12:45–13:00	8	1	10	1	
53	13:00–13:15	3		9		52
54	13:15–13:30	10		4	1	
55	13:30–13:45	4		9	1	
56	13:45–14:00	7		6		
57	14:00–14:15	10		4		47
58	14:15–14:30	6		6		
59	14:30–14:45	4		6		
60	14:45–15:00	8		3		
61	15:00–15:15	9		5		52
62	15:15–15:30	7		6		
63	15:30–15:45	5		8		
64	15:45–16:00	9		3		
65	16:00–16:15	3		9	2	49
66	16:15–16:30	8		6		
67	16:30–16:45	7		8		
68	16:45–17:00	6		2	1	
69	17:00–17:15	4		9		48
70	17:15–17:30	6		6	1	
71	17:30–17:45	7		5		
72	17:45–18:00	5		6		
73	18:00–18:15	7		6		52
74	18:15–18:30	8		5		
75	18:30–18:45	6		8		
76	18:45–19:00	6		6		
77	19:00–19:15	5		7		51
78	19:15–19:30	6		8		
79	19:30–19:45	6		5		
80	19:45–20:00	8		6		
81	20:00–20:15	4		8		51
82	20:15–20:30	8		8	2	
83	20:30–20:45	6		6		
84	20:45–21:00	4		7	1	
85	21:00–21:15	4		9		49
86	21:15–21:30	6		9		
87	20:30–21:45	5		6		
88	21:45–22:00	3		7		
89	22:00–22:15	4		8		48
90	22:15–22:30	3		9		
91	22:30–22:45	5		8		
92	22:45–23:00	2		9		
93	23:00–23:15	5		10		45
94	23:15–23:30	1		9		
95	23:30–23:45	1		9	1	
96	23:45–24:00	2		8		
	Total	509	11	513	16	1 022

From the observation data in Table 1, it can be seen that the total demand for all day arrivals and departures are 1022 flights, with arrival 513 flights and departure 509 flights. Cancelled flights due to airport capacity limitations are 27 flights, including arrival 16 flights and departure 11 flights. The actual total number of flights of the day is 995 flights, including arrival 497 flights and departure 498 flights. The On-time Performance rate is 97.36%. In other words, the cancellation rate of flights is 2.64%.

According to the definition of airport capacity, airport capacity is determined as the maximum number of operations that can be performed within a fixed time interval. This observation interval can be set to 15 minutes, 30 minutes, or 60 minutes. However, empirical data shows that intervals of 15 minutes can better reveal the fluctuations and changes in airport capacity in more detail based on the operational characteristics of a certain airport. Therefore, under the given conditions of a given airport and without affecting generality, this work uses observation data every 15 minutes to construct a capacity envelope.

**Fig 1:**
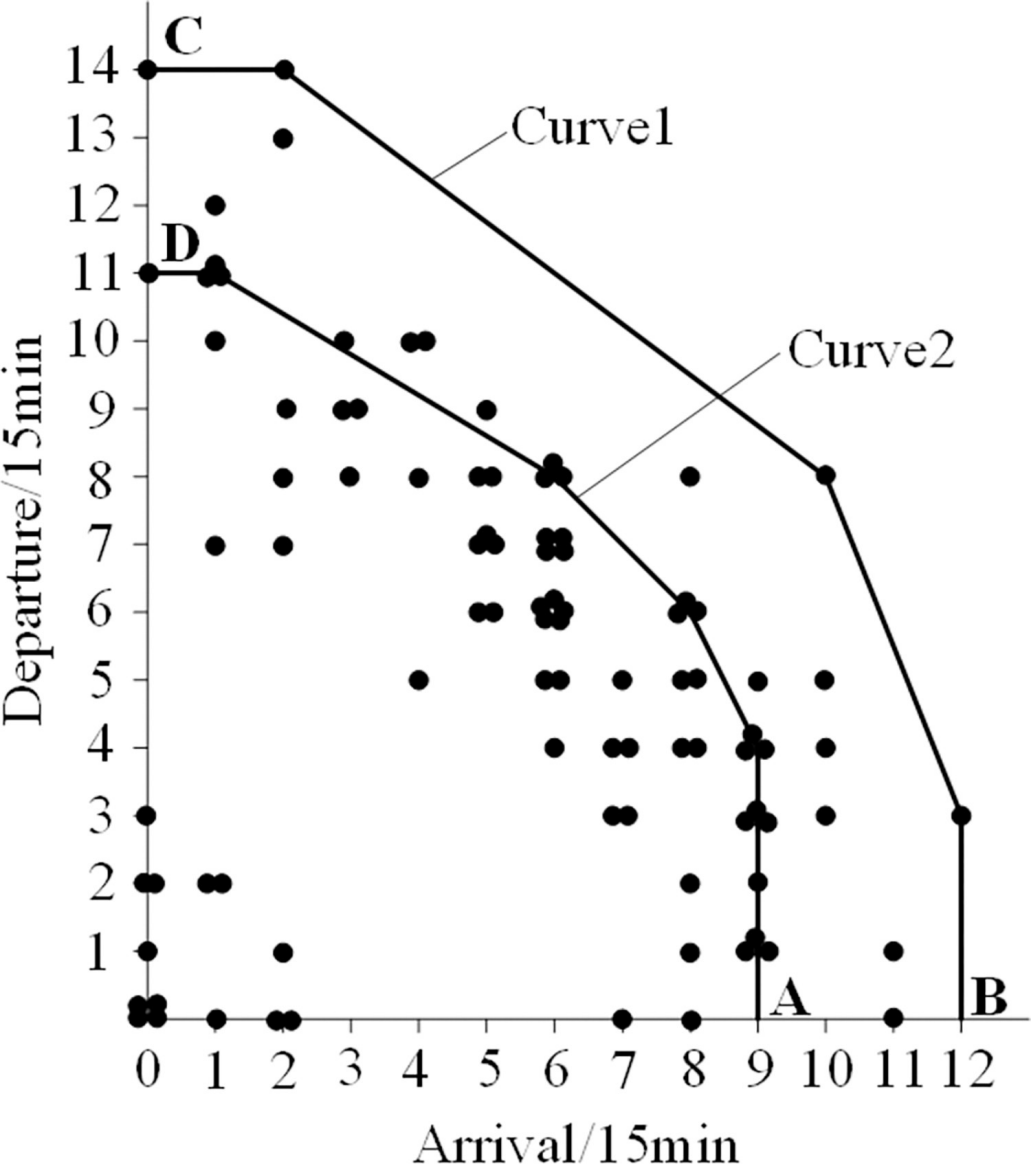
Distribution of data points and capacity envelope for arrival and departure. Curve 1-Ultimate capacity curve; Curve 2- Actual capacity curve.

On this basis, the authors come up with an empirical method as follows: In Table 1, the duration (from 0:00 to 24:00) is divided into 96 time periods, each with a length of 15 minutes. Each time slot corresponds to a total of 96 arrival and departure flight data pairs. Place all data into a coordinate system, with the horizontal axis representing the demand for flights arriving within 15 minutes and the vertical axis representing the demand for flights departing within 15 hours. Then, connect the relevant data points reasonably and generate the airport capacity curve, as shown in Fig 1.

The curve 1 in Fig 1 is a convex curve formed by connecting four points with arrival and departure demand frequency of 1, as well as points B and C, which imposes restrictions on other data points. Therefore, the points mentioned above can be considered as extreme values, and then the convex curve can be considered as the limit capacity curve of the airport. The vertex coordinates on a convex curve are (0,14), (2,14), (10,8), (12,3), and (12,0). This indicates that the maximum arrival capacity is 10 flights every 15 minutes, while the maximum departure capacity is 8. The maximum limit capacity (arrival+departure) is 18 flights per 15 minutes, while the maximum limit capacity is 72 flights per hour.

**Fig 2:**
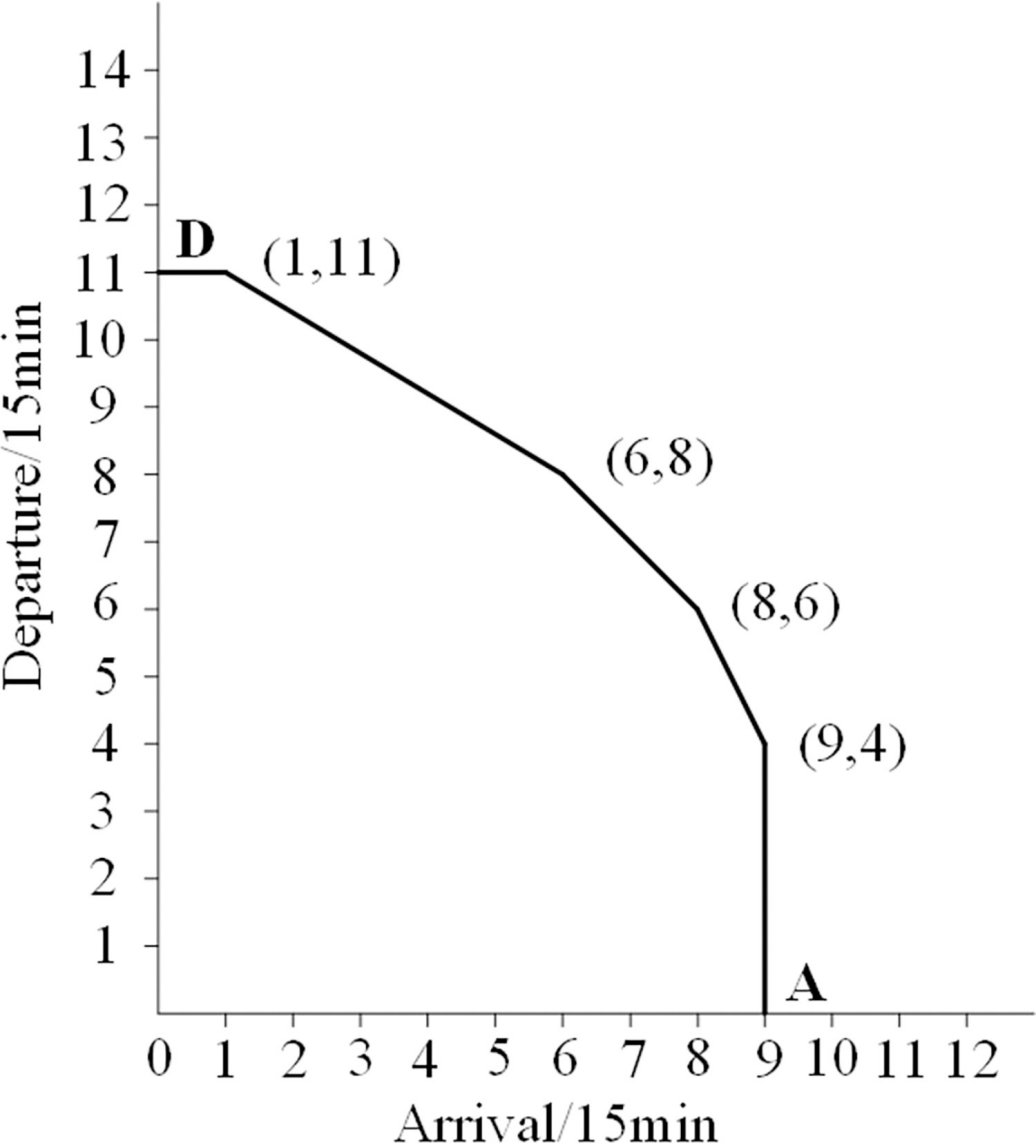
Airport actual capacity curve.

Curve 2 is a convex curve formed by connecting six points with arrival and departure frequencies of 3, including points A and D. Compared to curve 1, it is evident that curve 2 is relatively stable. Therefore, it is reasonable to use it as the actual airport capacity curve. The characteristics of curve 2 are shown in Fig 2.

From Fig 2, it can be seen that the coordinates of the six vertices on the convex curve are (0,11), (1,11), (6,8), (8,6), (9,4), and (9,0), respectively. This indicates that the actual arrival capacity is 8 flights every 15 minutes, the actual departure capacity is 6 or the arrival capacity is 6, and the departure capacity is 8 flights every 15 minutes. The actual capacity (arrival+departure) is 14 flights per 15 minutes, and the maximum actual capacity (arrival+departure) is 56 flights per hour.

Given that airport capacity is typically represented by airport capacity curves. The airport capacity curve possesses convex function properties and satisfies linear constraint capabilities. Then, the convex feasible area formed by the closure of the airport capacity curve and the horizontal and vertical axes of the coordinate system is the airport capacity range, and the feasible area can be considered the airport capacity.

### 3.2 Initial traffic demand

**Fig 3:**
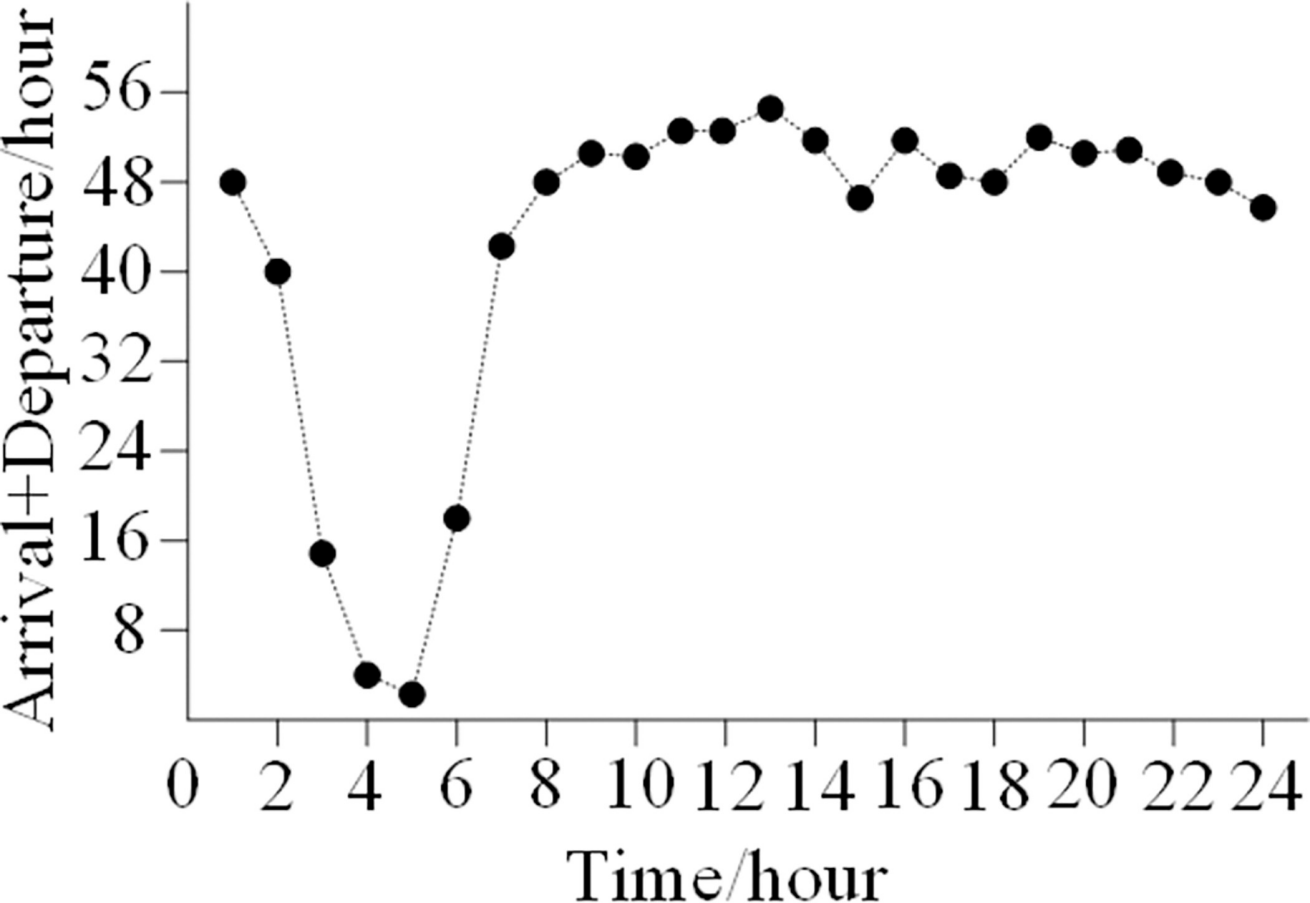
Traffic demands of arrival + departure from 0:00 to 24:00.

Fig 3 shows the daily in-and-out capacity demand. As the figure illustrates, the hours of 07:00 to 24:00 are when daily arrival and departure capacity is most in-demand. The average demand for arrival and departure capacity is 50.2 flights per hour, while the demand for arrivals and departures is 45–54 flights per hour.

**Fig 4:**
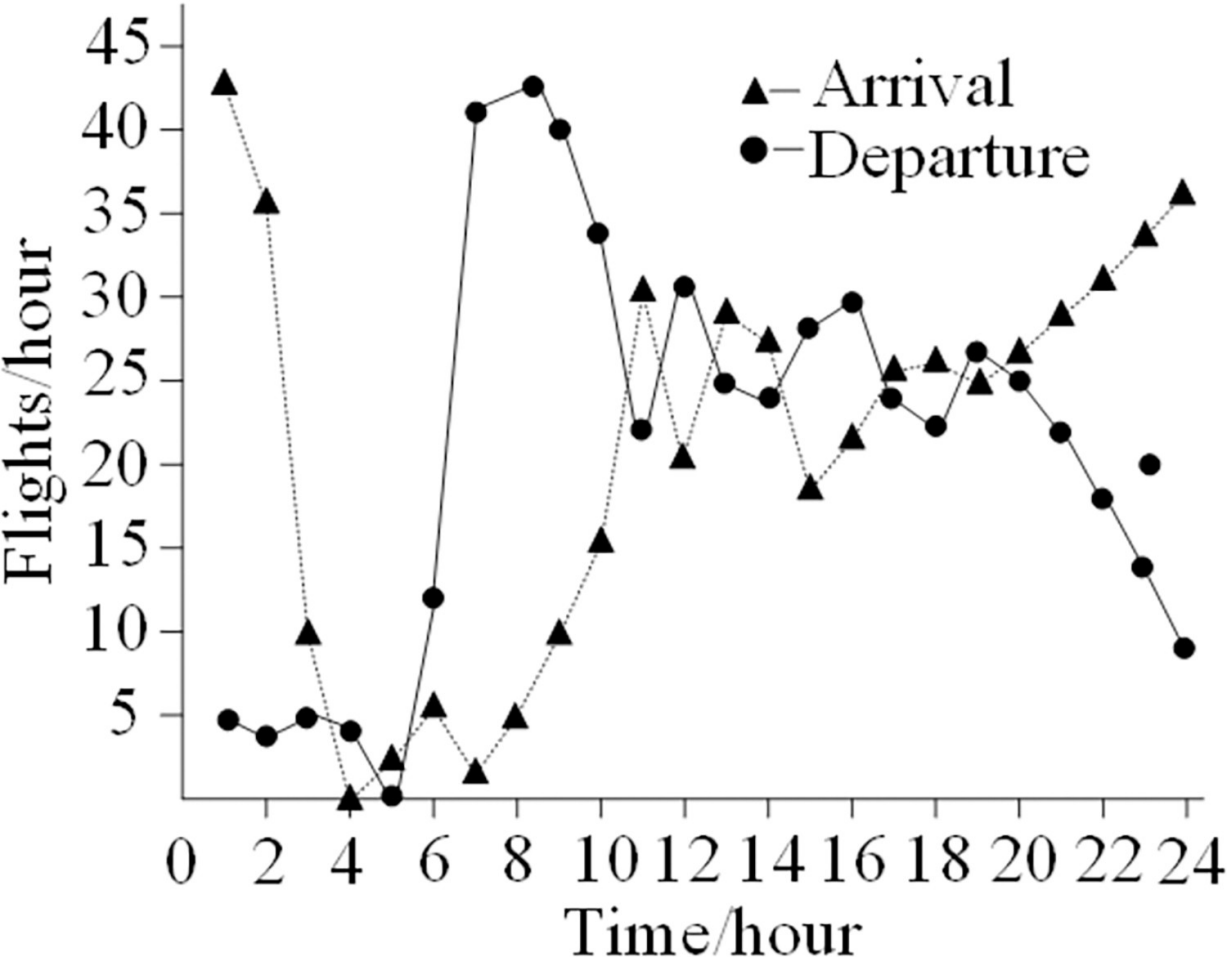
Traffic demands of arrival and departure from 0:00 to 24:00.

Fig 4 was created to further show how the capacity requirements changed from 0:00 to 24:00. The findings show that the growth and decline characteristics of the demand for arrival and departure capacity are opposite. For example, the hourly demand for arrival flight capacity decreases from a peak of 43 flights to 0 flights, then gradually increases to 36 flights. The peak demand for arrival flight capacity occurs between 1:00 and 2:00. Between 7:00 and 9:00 is when demand for originating flights peaks, after which capacity demand gradually declines to 9 flights per hour from 5 flights per hour to 0. During the period from 10:00 to 20:00, the capacity demand for arriving and departing flights during the same time period is basically equal, and the traffic demand for arriving and departing flights is between 20 and 30 per hour.

In addition, it should be noted that from 10:00 to 20:00, the average demand for departure and arrival is as high as 50.2 flights per hour, which is prone to traffic congestion and flight delays. Therefore, the demand for arrival and departure capacity should be optimized to eliminate the impact of congestion and delay.

### 3.3 Collaborative optimization model

Because of the convex function property and piecewise linearity of the airport capacity curve, this set of envelope segments can actually be viewed as a collection of functional inequalities. This collection of functional inequalities is actually the constraint that regulates the airport’s capacity for arrivals and departures. On this basis, airport capacity optimization is an optimal control problem with criteria, and constrained optimization problems can be treated as an integer linear programming problem by utilizing the convexity and piecewise linearity of the capacity curve. Therefore, without considering the capacity limit of the positioning point, the optimized objective function is represented as follows:

The problem is formulated using the following fundamental notations:

*T =* a time interval of interest consisting of *N* time slots of length Δ (e.g., Δ = 15 minutes); *T* = *N*Δ;

*I* = {1, 2,…, *N*} = a set of time slots, the time slots of each group are 15 minutes long;

*u*_*i*_ = airport arrival capacity at the *i*th time slot, *i*∈*I*;

*v*_*i*_ = airport departure capacity at the *i*th time slot, *i*∈*I*.

Where *u*_*i*_ and *v*_*i*_ are integers, because the number of flights can only be counted as integers.

α = a priority ratio. Specifically, it is a variable priority parameter. The value range is 0≤α≤1. If α = 0.5, it indicates that arrival and departure have equal priority. For airport flow control, for arrival and departure, α = the optimal constant capacity of 0.5 is the same. If 0.5< α≤ 1, then it means that the arrival flight has priority over the departure flight. On the contrary, departure flights are actually the preferred choice. By changing parameter α, traffic flow controllers will be able to generate different airport capacity allocation strategies and choose any alternative solution based on reasons other than mathematical form.

The two arrival priorities (α = 0.5 and 0.7) are the ideal capacity values, as demonstrated by the actual operation experience. A complete solution to the departure problem is provided by the optimal strategy when α = 0.5, and a complete solution to the arrival problem is provided when α = 0.7. But when α = 0.7, or when the arrival priority is raised from 0.5 to 0.7, the quantity of delayed arrivals drops dramatically. Thus, for the joint optimization of airport capacity and traffic demand, α = 0.5 and 0.7 are investigated in this work.

The objective function of the optimization problem is as follows (1):

$$max\sum_{i=1}^{N}(N-i+1)[\alpha u_{i}+(1-\alpha )v_{i}],\;0\leq \alpha \leq 1$$ (1)

Subject to

$$0\leq u_{i}\leq 9,\;i=1,2,\cdots ,N$$ (2)

$$0\leq v_{i}\leq 11,\;i=1,2,\cdots ,N$$ (3)

$$v_{i}+0.6u_{i}\leq 11.6,\;i=1,2,\cdots ,N$$ (4)

$$v_{i}+u_{i}\leq 14,\;i=1,2,\cdots ,N$$ (5)

$$v_{i}+2u_{i}\leq 22,\;i=1,2,\cdots ,N$$ (6)

Functional inequality serves as an expression for the constraints. The constraints limiting the excessive demands are these functional inequalities, which also have a significant role in limiting the demands for the arrival and departure flow at airports. Each functional inequality is obtained by mathematical calculation according to the coordinates of the envelope line in Fig 2. The number of functional inequalities is determined by the number of vertices on the convex curve.

In this set of functional inequalities:

9 in Eq (2) is the maximum value of arrival flights on the abscissa;

11 in Eq (3) is the maximum value of departure flights on the ordinate;

11.6, 14, and 22 are the values corresponding to functional inequalities (4), (5), and (6), which are obtained through mathematical calculations. 0.6, 1, and 2 are the coefficient values of *u*_*i*_ corresponding to function inequalities (4), (5), and (6), which are also obtained through mathematical calculations.

Based on the actual arrival and departure capacity demands of the airport, a 10:00–12:00 time interval with heavy traffic and the most prone to traffic congestion and flight delays is selected as an example to study the arrival and departure capacity optimization. The time interval is 15 minutes, which is divided into 8 slots (see Table 2).

**Table 2:** Initial traffic demand and delay.

Slot /15min	Initial traffic demands		Queue waiting	
Start End	Arrival	Departure	Arrival	Departure
10:00–10:15	5	6	0	0
10:15–10:30	10	4	1	0
10:30–10:45	7	4	0	0
10:45–11:00	9	8	0	0
11:00–11:15	7	3	0	0
11:15–11:30	6	8	0	0
11:30–11:45	6	6	0	0
11:45–12:00	2	14	0	3
Total	52	53	1	3

**Fig 5:**
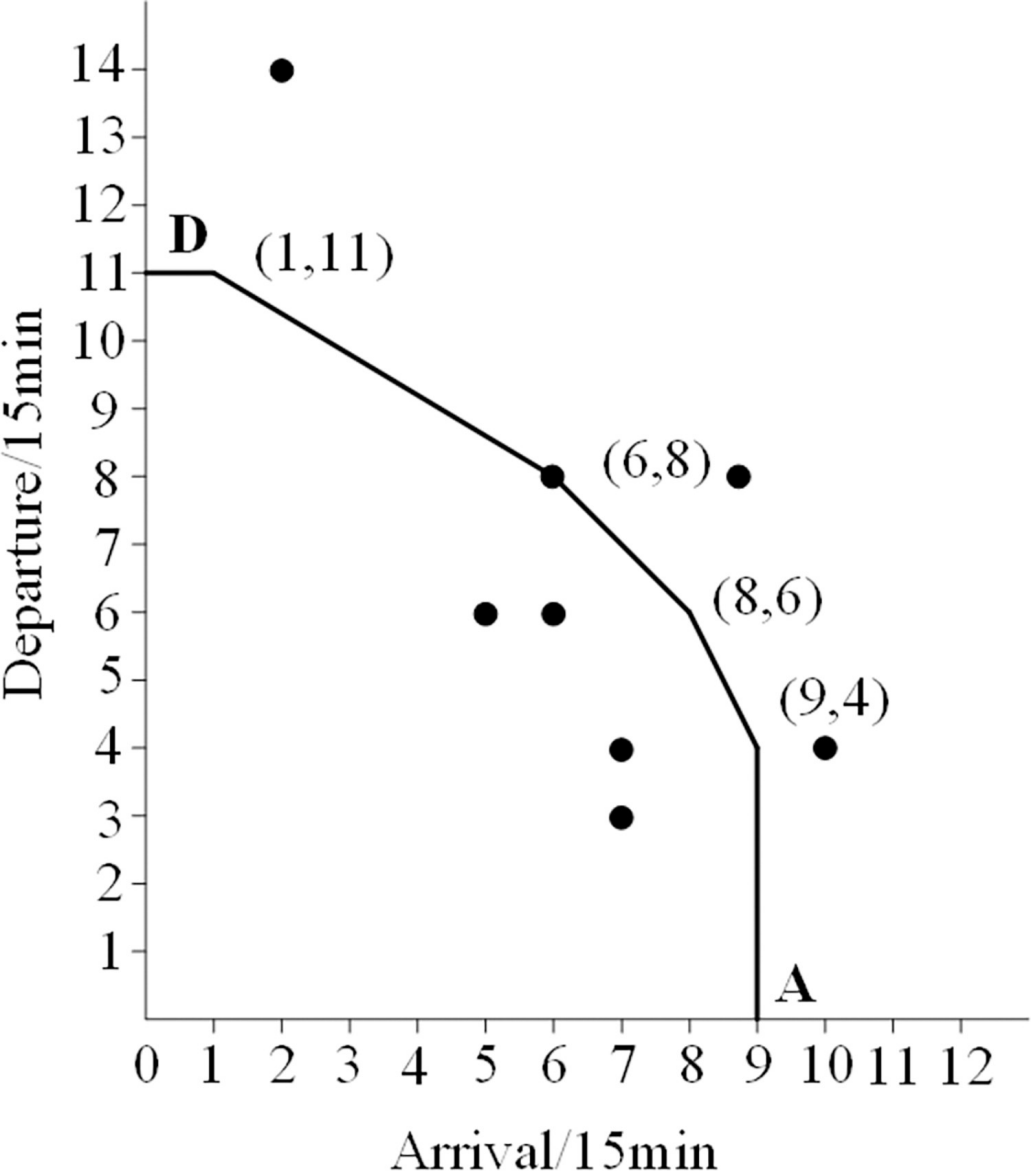
Distribution of initial traffic demands before optimization.

In the 2-hour time interval, the total demand for arrivals and departures is 105 flights, including 52 arrival flights and 53 flights required to take off. However, according to the actual capacity of the airport every 15 minutes, a total of 4 arrival and departure flights in two slots exceed the actual capacity of the airport (see Fig 5), and delays are inevitable. It was found that 1 arrival flight was delayed from 10:15 to 10:30, and 3 departure flights were delayed from 11:45 to 12:00.

### 3.4 Optimization algorithm

Numerous optimization algorithms have emerged in recent years and have been preferred to address different optimization problems, including those in the field of air traffic management. The classic Genetic Algorithm (GA) was used by Xiaobing Hu and Ezequiel Di Paolo [15] in their work, concerning the scheduling and sequencing problem of aircraft arrivals without considering much the departures in a multi-runway system. In the research by Lucio Bianco and Maurizio Bielli [16], potential optimization models and solution algorithms were discussed and a proposal for a multilevel air traffic management model was presented. The functions associated with flow control, flight strategic control, and aircraft sequencing in a terminal area were also examined. However, when it came to the capacity issue, there was a lack of thought to reveal the dynamic relationships between arrivals and departures. In some other optimization research [17–19], the shortcomings of the classic GA algorithm in some practical applications have been revealed. Zhai Longzhen and Feng Shaohong [20] proposed an evacuation route planning method based on their suggested optimization algorithm, which showed advantages rivaling the classic GA in experimental comparison.

On this basis, a modified GA presented in this work to solve the proposed capacity estimation model in this work with the goal of collaboratively optimizing the airport capacity in order to balance supply and demand and avoid air traffic congestion.

The parameters of the improved genetic algorithm are as follows:

Initial population size is 100;

Maximum generations is 100;

Selection rate is 0.2;

Crossover rate is 0.7;

Variation rate is 0.1.

Accumulated initial demand values for each time slot arrivals and departures:

*u*_*i*_ = [5;15;22;31;38;44;50;52], accumulated value of initial demands for arrival flights at each time slot;

*v*_*i*_ = [6;10;14;22;25;33;39;53], accumulated value of initial demands for departure flights at each time slot;

α = 0.5; α = 0.7, (α is between 0 and 1.)

**Fig 6:**
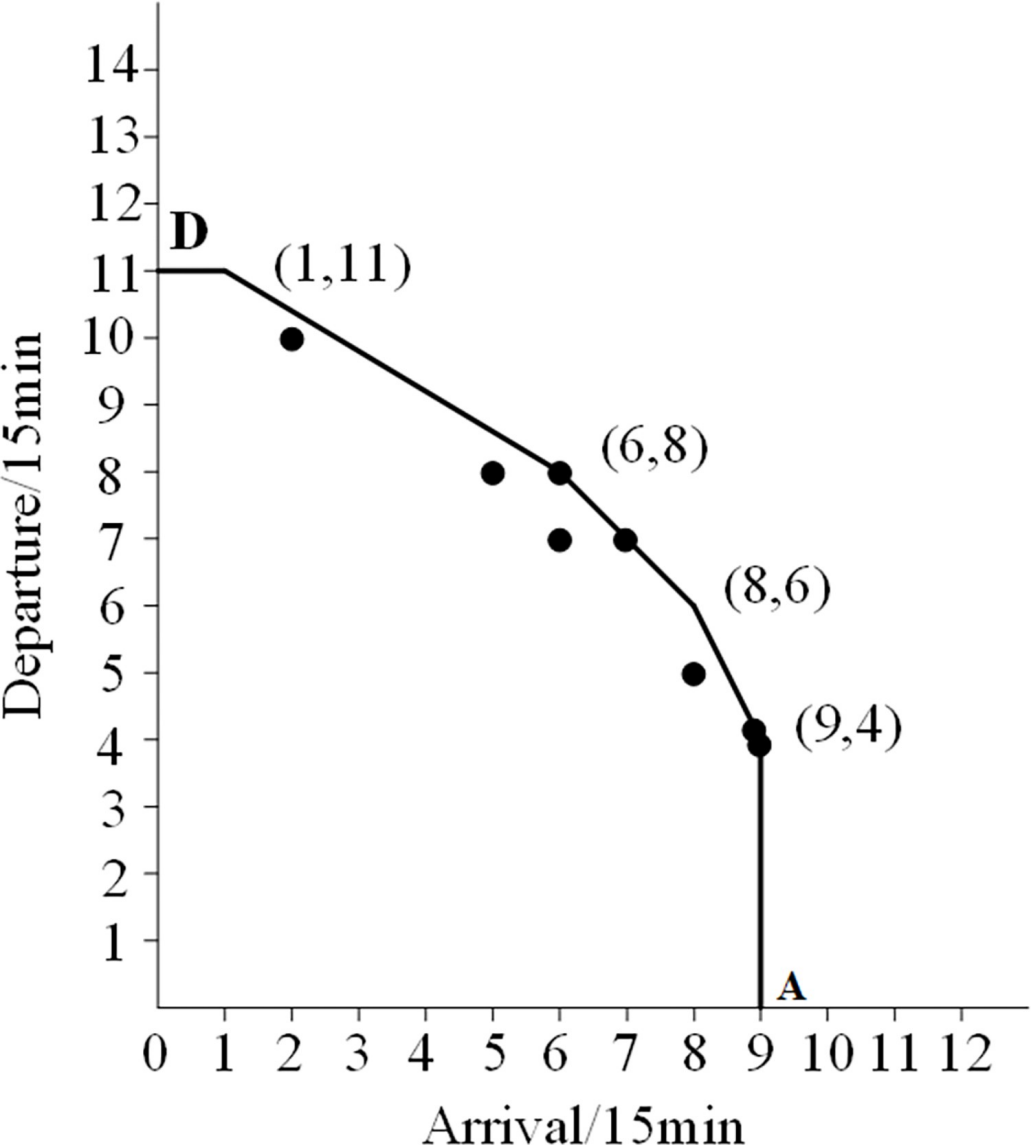
Distribution of traffic demands after optimized with priority (α = 0.5).

**Fig 7:**
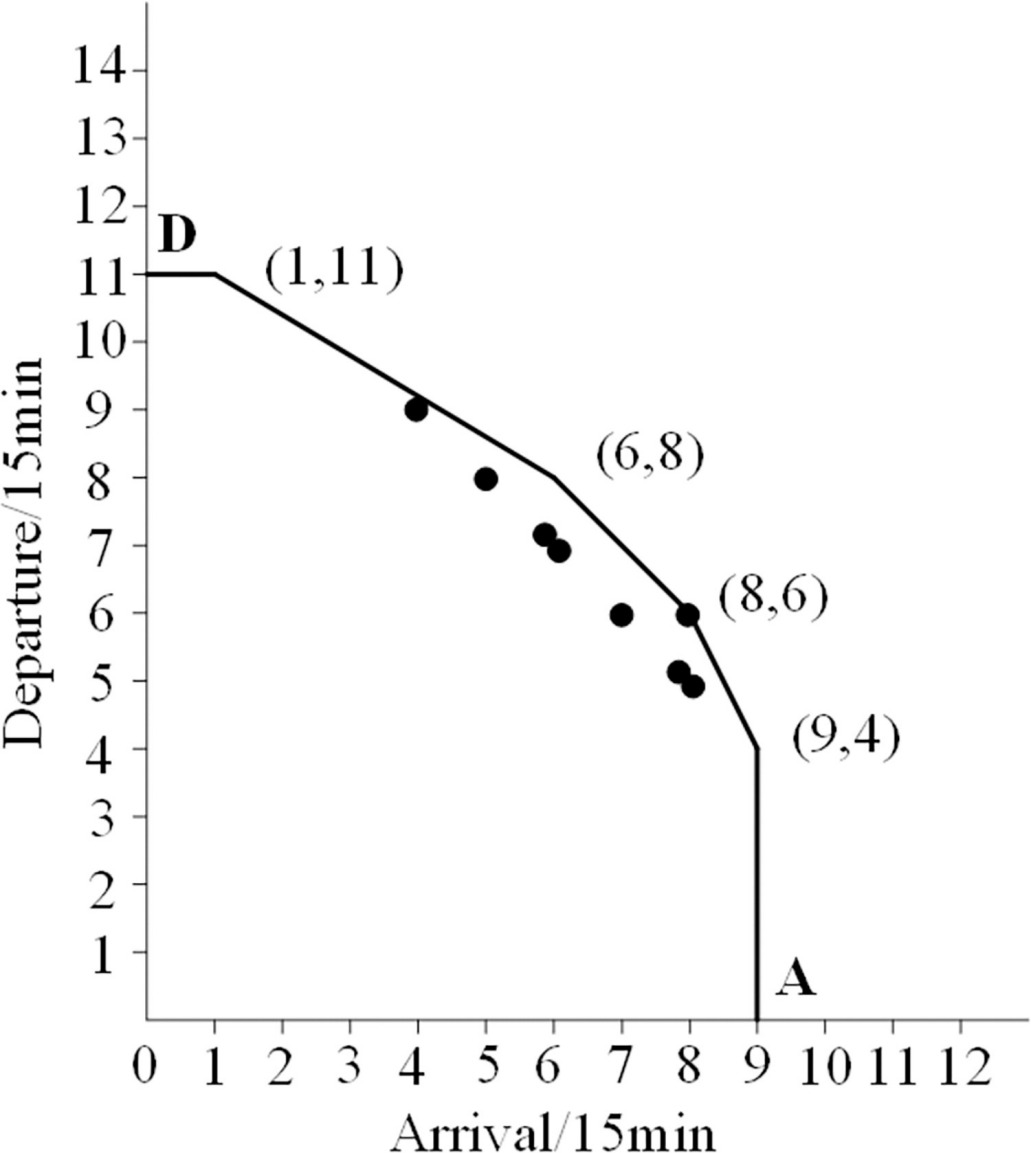
Distribution of traffic demands after optimized with priority (α = 0.7).

The priority ratio is set to α = 0.5 based on the flow demands of arrivals and departures, indicating that arrivals and departures share the same priority. Table 3 and Figs 6 and 7 show that all of the flow demands for arrival and departure meet the airport capacity constraints. After conducting capacity optimization, the flow demands for arrivals and departures are redistributed so that the supply and demand achieves balanced, thus the risk of congestion and delay is reasonably avoided, assuming that the demands for arrival and departure remain unchanged. In contrast, when α = 0.5, it benefits the departure demands. There are 10 flights departed from 11:45–12:00, but only 2 arrived. When α = 0.7, or the arrival flight’s priority ratio is increased by 0.2, the optimal strategy favors the arrival, narrowing the gap between arrivals and departures capacity demands in each slot.

**Table 3:** Results of optimized with priority.

Slot/15min	Optimized with priority α = 0.5		Optimized with priority α = 0.7	
Start End	Arrival	Departure	Arrival	Departure
10:00–10:15	5	8	5	8
10:15–10:30	9	4	6	7
10:30–10:45	8	5	7	6
10:45–11:00	7	7	8	6
11:00–11:15	9	4	8	5
11:15–11:30	6	8	6	7
11:30–11:45	6	7	4	9
11:45–12:00	2	10	8	5
Total	52	53	52	53

Table 4 shows the comparison results between the initial flight demands and the optimized flight demands in each slot.

**Table 4:** Comparison of optimized results of traffic demands.

Slot/15min	Initial traffic demands	Optimized with priority α = 0.5	Optimized with priority α = 0.7
Start End	Arrival + Departure	Arrival + Departure	Arrival + Departure
10:00–10:15	11	13	13
10:15–10:30	14	13	13
10:30–10:45	11	13	13
10:45–11:00	17	14	14
11:00–11:15	10	13	13
11:15–11:30	14	14	13
11:30–11:45	12	13	13
11:45–12:00	16	12	13
Differences	7	2	1

The grade difference of the initial demands in each time slot is 7 (17–10 = 7). When the priority parameter is set to α = 0.5, after the initial flight capacity demands are optimized, the flight demand grade difference is reduced to 2 (14–12 = 2). When the priority is set to α = 0.7, after optimization, the difference in demand is only 1 (14–13 = 1), which minimizes the flight demand grade difference in each slot.

## 4. Results and discussions

### 4.1 Examples and validations

To verify the effectiveness and feasibility of the suggested method of this work, the peak hours of 12:00–15:00 were chosen as an example. Table 5 shows the arrival and departure demands in these time slots.

**Table 5:** Initial traffic demand and delay.

Slot/15min	Initial traffic demands		Queue waiting	
Start End	Arrival	Departure	Arrival	Departure
12:00–12:15	4	5	0	0
12:15–12:30	6	7	0	0
12:30–12:45	9	5	0	1
12:45–13:00	10	8	1	3
13:00–13:15	9	3	0	0
13:15–13:30	4	10	0	1
13:30–13:45	9	4	0	0
13:45–14:00	6	7	0	0
Total	57	49	1	5

**Fig 8:**
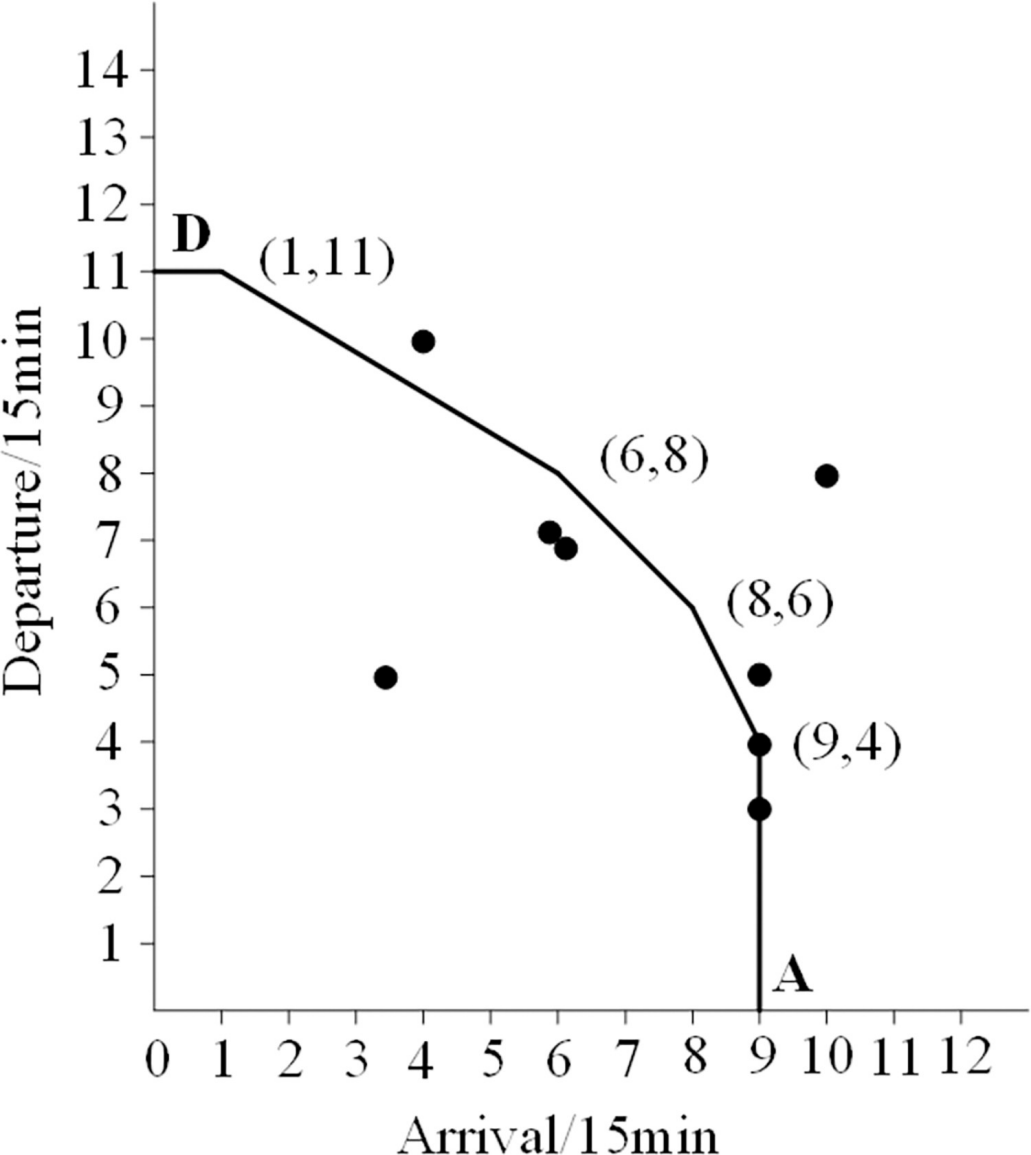
Distribution of initial traffic demands before optimization.

The total demand for arrival and departure flights during the 2-hour interval is 106. Specifically, 57 flights arrive and 49 flights depart. However, there were 6 flights that exceeded the airport’s actual capacity, resulting in delays (see Fig 8). 1 arrival was delayed between 12:45 and 13:00, 3 departure flights were delayed between 12:45 and 13:00, and 1 departure flight was delayed between 13:15 and 13:30.

**Fig 9:**
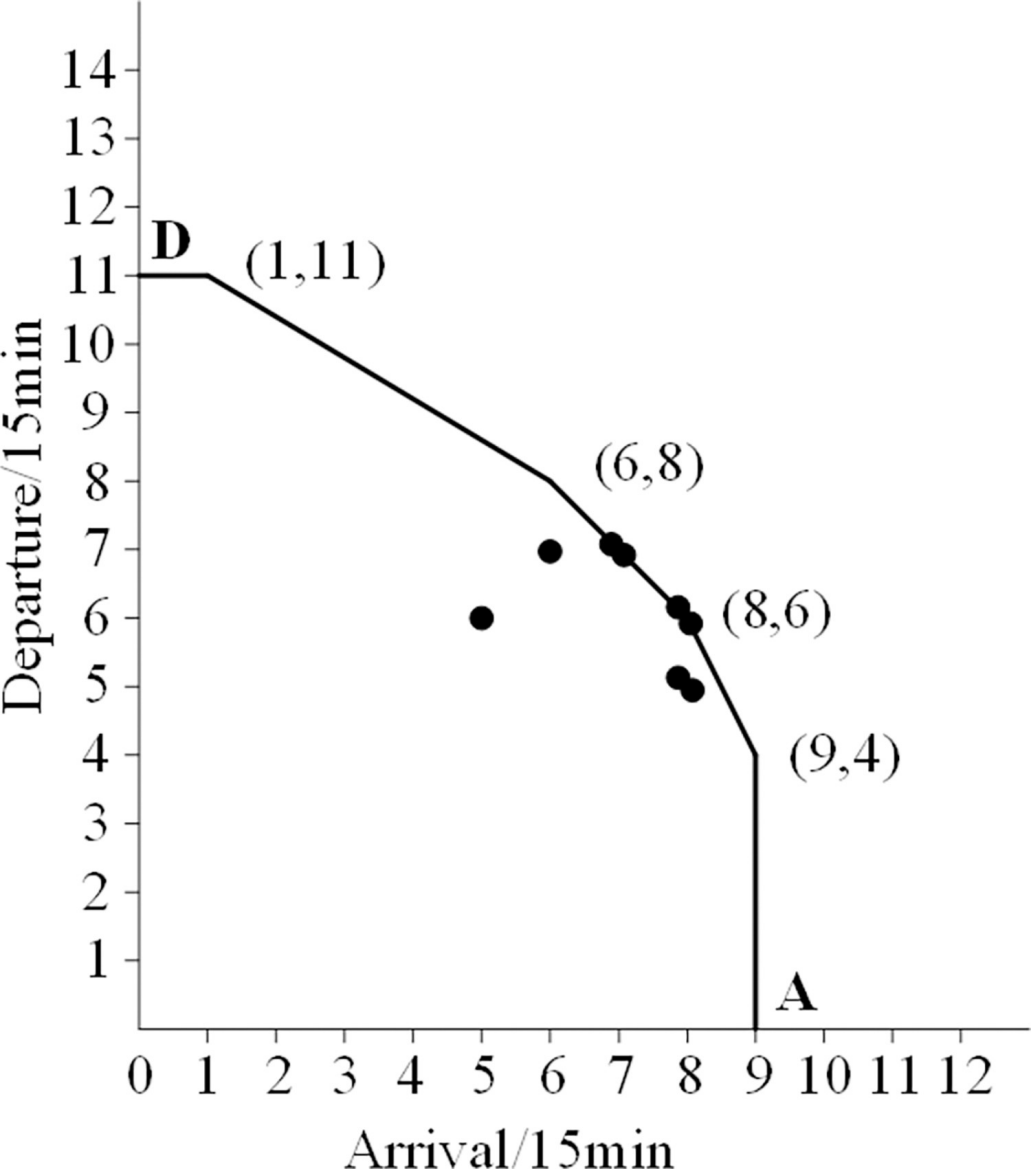
Distribution of traffic demands after optimized with priority (α = 0.5).

When the priority ratio is set to 0.5, the weight of arrivals and departures is equal. The results are shown in Table 6 and Fig 9. As can be observed, the optimization results for the priority ratio α = 0.5 demonstrate that, in the case of constant arrival and departure demands, 6 flights’ arrival and departure demands can be met by collaborative optimization while also reasonably avoiding the risks of congestion and delays.

**Table 6:** Results of optimized with priority.

Slot /15min	Optimized with priority α = 0.5		Optimized with priority α = 0.7	
Start End	Arrival	Departure	Arrival	Departure
12:00–12:15	5	6	6	5
12:15–12:30	6	7	6	7
12:30–12:45	8	5	8	5
12:45–13:00	8	6	8	6
13:00–13:15	8	5	8	5
13:15–13:30	7	7	7	7
13:30–13:45	7	7	7	7
13:45–14:00	8	6	7	7
Total	57	49	57	49

**Fig 10:**
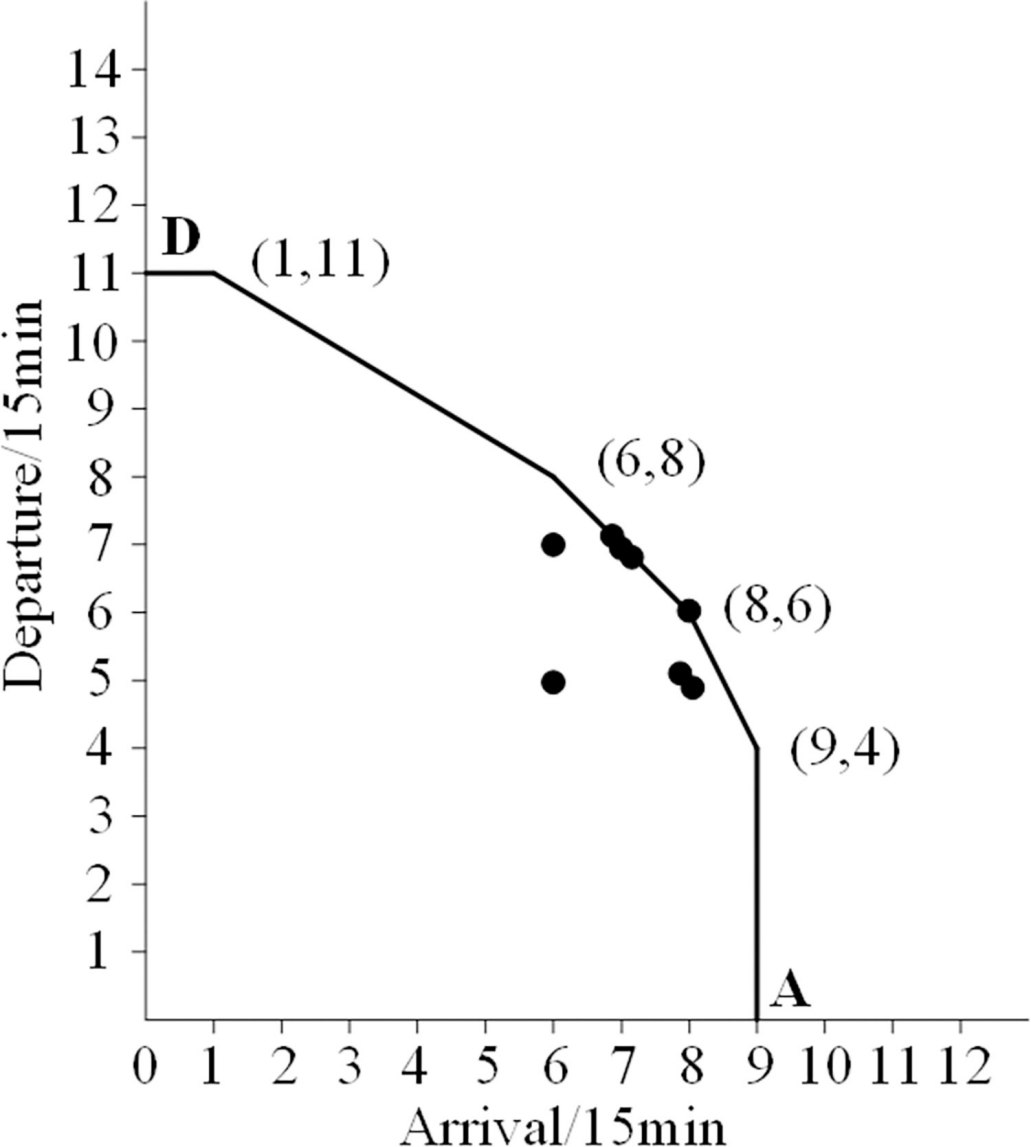
Distribution of traffic demands after optimized with priority (α = 0.7).

If the priority ratio is set to 0.7, then the optimal strategy favors arrival flights. The 6 arrivals, as indicated in the table, satisfy the airport capacity constraints likewise following cooperative optimization of capacity demands, then no delays occur under this circumstance. Results are shown in Table 6 and Fig 10.

Table 7 compares the capacity demand level of each slot following collaborative optimization to its initial flight demands. The table shows that there are 9 flights (18–9 = 9) in the initial flight demand grade difference for each slot. After the initial flight capacity demands are optimized, the flight demand grade difference is reduced to 3 flights (14–11 = 3) when the priority is set to α = 0.5. The flight demand grade difference is 3 flights (14–11 = 3) when the priority is set to α = 0.7 following optimization. It is clear that the anticipated result has been achieved as both arrivals and departures share the same grade difference.

**Table 7:** Comparison of optimized results of traffic demands.

Slot /15min	Demand range	Optimized with priority α = 0.5	Optimized with priority α = 0.7
Start End	Arrival + Departure	Arrival + Departure	Arrival + Departure
12:00–12:15	9	11	11
12:15–12:30	13	13	13
12:30–12:45	14	13	13
12:45–13:00	18	14	14
13:00–13:15	12	13	13
13:15–13:30	14	14	14
13:30–13:45	13	14	14
13:45–14:00	13	14	14
Total	9	3	3

Flights were canceled because the initial capacity distribution did not accommodate traffic demand. The case validation result shows that all canceled flights within each time slot have satisfied the airport capacity constraints upon applying the proposed optimization method. It demonstrates that once requirements and supply schemes have been effectively optimized, cancellations can be properly avoided. This further proves that the core of the strategy proposed in this work is to collaboratively adjust supply and demand rather than simply relying on expanding airport scale, which assists in achieving the goal of maximizing the utilization of existing capacity resources to meet traffic needs.

### 4.2 Results

The above research methods and case validation results show that the Flight On-time Performance rate can be lifted by 6% and delays can be technically avoided by applying a reasonable capacity distribution scheme. This indicates that the effective use of airport historical operating data is the key to obtaining the actual and ultimate capacity of the airport, as these data themselves reflect the characteristics and comprehensive operational capabilities of the airport. The method of constructing airport capacity curves using the envelope method and historical airport capacity operation data is effective because it provides convex function features and piecewise linearity, making the capacity optimization problem reformed as an optimization control problem with criteria. In addition, using the Integer Linear Programming (ILP) method to construct a collaborative optimization mathematical model for airport capacity and arrival and departure demand has certain advantages when solving specific problems. The proposed model views arrival and departure as interdependent processes, and it uses a series of functional inequalities to limit airport capacity based on arrival and departure flows. These functional inequalities play a role in limiting excessive demand and redistributing initial arrival and departure traffic flow. Put differently, it only reallocates time slots without reducing its initial traffic demand. Without increasing airport capacity, this three-in-one collaborative optimization approach can fully utilize current capacity resources to meet transportation demands. Thus, it enhances operational quality and efficiency, lowers the possibility of air traffic congestion and delays, and greatly lessens the difficulty of air traffic scheduling while also optimizing the interests of airlines. Furthermore, the enhanced genetic algorithm by improving fitness functions, selection, crossover, and mutation operators showcased in this study differs from traditional genetic algorithms in that it possesses a reasonable capacity for solving models based on results. The optimal solution obtained by this algorithm is not unique but multiple and optimal, which is preferred and desired. Therefore, this model can serve as effective decision support for air traffic flow controllers, as by changing the priority settings of the model, the optimal flow allocation solution can be more dynamically selected and formulated under various circumstances.

The merit of this proposed method is that it fully considers the characteristics of the airport and actual operational data(see S1 File), which reflect the comprehensive features of the airport, and thus it creates a supply-demand model to reveal the internal mechanism of supply and demand interactions. This approach is more suited for developing flight time allocation schemes in the tactical real-time traffic management phase to prevent congestion and delays brought on by the effects of processing efficiency in the strategic traffic management phase. Especially in optimizing the response to potential excess demand, such as when alleviating peak flight demand during particular periods of time like holidays, more effective time slot scheduling schemes can be provided. Therefore, it has potential value for use at large airports.

## 5. Conclusions

This study examines critical aspects of airport capacity and slot allocation, concerning actual capacity estimation, collaborative optimization modeling, and algorithm solving.

1. An empirical method along with an analytical approach is proposed, constructing a capacity envelope to estimate the actual capacity by using airport observation data. The capacity curve defines the capacity feasible area that covers the full range of airport operation restrictions for arrival and departure under different circumstances. The convex function and segmented features of the capacity envelope curve make it fundamental and feasible to reformulate the capacity optimization problem into an optimization control problem with criteria.
2. A collaborative capacity optimization model was created with the capacity envelope’s convex function properties and piecewise linearity, the capacity-demand relationship, and a number of functional inequalities serving as model constraints and priority parameters. It can be used to handle excessive demand during peak periods.
3. An enhanced optimization algorithm has been introduced as a means of solving the proposed model. By varying the model parameters, the suggested model and solution algorithm can be used to generate the appropriate strategic flow allocation plan in a flexible manner, as shown by the example validation results. This makes them useful ways for air traffic flow controllers to make decisions.

The method presented in this research(see S2 File) will assist other hub airports in solving similar problems. It is beneficial for maximizing available capacity, properly estimating the airport’s practical capacity, balancing supply and demand, increasing operational throughput and efficiency, and lowering the risk of traffic congestion and delays.

## Supporting information

S1 File Data availability. (PDF)

S2 File Highlights. (PDF)
