# Supplementary material for: Collaborative optimization model and algorithm for airport capacity and traffic flow allocation
Source: PLoS One. 2024 Mar 22;19(3):e0298540. doi: 10.1371/journal.pone.0298540 (PMC10959351; doi:10.1371/journal.pone.0298540)
Supplement: S1 File — (PDF) [file pone.0298540.s001.pdf]

# Data availability

The data collected and used in this paper are obtained from CAAC Southwest Air Traffic Administration and the Chengdu Shuangliu International Airport of China. The data is authentic and public, for daily flight information and the Departure and Arrival status please visit the website https://www.cdairport.com/en/dynamic3.aspx?t=127.

This study only involves computer simulations. Codes and sources used in our work are available at OSF: https://osf.io/4mtzu
