# Supplementary material for: Collaborative optimization model and algorithm for airport capacity and traffic flow allocation
Source: PLoS One. 2024 Mar 22;19(3):e0298540. doi: 10.1371/journal.pone.0298540 (PMC10959351; doi:10.1371/journal.pone.0298540)
Supplement: S2 File — (PDF) [file pone.0298540.s002.pdf]

Highlights

- An empirical method combined with an analytical approach is proposed, constructing a capacity envelope to estimate the actual capacity by using airport observation data.
- A collaborative capacity optimization model was created with the capacity envelope's convex function properties and piecewise linearity, the capacity-demand relationship, and a number of functional inequalities serving as model constraints and priority parameters.
- An improved genetic algorithm was proposed to solve the model.
